# Catestatin in Cardiovascular Diseases

**DOI:** 10.3390/ijms26062417

**Published:** 2025-03-07

**Authors:** Joanna Kulpa, Jarosław Paduch, Marcin Szczepanik, Anna Gorący-Rosik, Jakub Rosik, Magdalena Tchórz, Andrzej Pawlik, Jarosław Gorący

**Affiliations:** 1 Department of Physiology, Pomeranian Medical University, 70-111 Szczecin, Poland; joanna.h.kulpa@gmail.com (J.K.); jaroslawpaduch@hotmail.com (J.P.); marcin.t.szczepanik@gmail.com (M.S.); magdalena.tchorz98@gmail.com (M.T.); pawand@poczta.onet.pl (A.P.); 2 Independent Laboratory of Invasive Cardiology, Pomeranian Medical University, 70-111 Szczecin, Poland; ania.goracy@gmail.com (A.G.-R.); jaroslaw.goracy@pum.edu.pl (J.G.)

**Keywords:** arterial hypertension, cardiovascular disease, catestatin, coronary artery diseases, heart failure, molecular mechanisms

## Abstract

Cardiovascular diseases are one of the leading causes of mortality and morbidity worldwide. The pathogenesis of this group of disorders is highly complex and involves interactions between various cell types and substances, among others, catestatin (CTS). In recent years, numerous researchers have expanded our knowledge about CTS’s role in development and its potential for the treatment of a variety of diseases. In this review, the authors discuss CTS’s importance in the pathogenesis of arterial hypertension, coronary artery disease, and heart failure. Moreover, we present CTS’s influence on heart and vessel function.

## 1. Introduction

Cardiovascular diseases (CVDs) account for one of the leading causes of morbidity and mortality worldwide. As CVDs remain the dominant cause of death in Europe, ischemic heart disease (IHD) has been the leading reason [1]. IHD is a group of related syndromes exhibiting myocardial ischemia and dysfunction due to an imbalance between the supply and the heart’s demand for blood. In most cases, atherosclerosis is the main factor contributing to stenosis in the coronary arteries and, therefore, a reduction in blood flow [2,3]. Atherosclerosis is a chronic inflammatory disease in which cholesterol-rich plaques deposit inside the arterial walls. A complex multicellular process characterizes it. Both genetic and environmental factors facilitate the expansion of the atherosclerotic plaques in various arterial territories [4]. Several risk factors for IHD have been established, including hypercholesteremia, diabetes, arterial hypertension (HA), obesity, and metabolic syndrome [5]. Developing and implementing novel preventive and treatment methods should be crucial to mitigate CVD risk factors and reduce the public health burden of CVDs in all countries. Chromogranin A (CgA) is a pro-protein found in neuroendocrine organs, more precisely, in the secretory granules of chromaffin cells. Proteolytic cleavage of CgA generates several biologically active peptides, such as pancreastatin, an inhibitor of glucose-induced insulin secretion; WE14, a peptide that is an antigen for diabetogenic CD4+ T-cell clones; serpinin, an adrenergic peptide; vasostatin a vasodilating, antiadrenergic, and antiangiogenic peptide, and catestatin (CTS) [6,7]. Based on the previous research, CgA and some of its bioactive fragments, CTS, among others, appear to act as a potential biomarker for various neoplastic and inflammatory diseases as well as CVDs [8,9,10,11]. CTS has emerged as a pleiotropic peptide, providing several cardioprotective effects. CTS may exhibit different functions by binding to various receptors and then causing subsequent activation of many signaling pathways [8,10,11].

Furthermore, previous data demonstrated that single nucleotide polymorphisms (SNPs) appearing in the CTS-expressing region of the *CHGA* gene might result in different variants of this molecule, which may exert different activity [8]. CTS’s cardiovascular effects include suppression of beta-adrenergic activation and consequently acting in a negative inotropic and chronotropic way, stimulating angiogenesis and proliferation of vascular smooth muscle cells, lowering endothelial thrombogenicity, and suppressing atherosclerosis and inflammation [8]. To this date, many studies have demonstrated the role of CTS in the pathogenesis and development of various CVDs, such as HA, diabetes mellitus (DM), atherosclerosis, and coronary heart disease [2,6]. Its levels appeared to also be different in heart failure (HF) [12]. Although there are a large amount of data from existing basic and clinical research, many mechanisms underlying CVD pathogenesis have yet to be explained. However, the role of CTS as a biomarker in this group of diseases is still conceptive. The most promising CTS measurement application seems to be in patients with HF [8]. It may deliver additive prognostic input to natriuretic peptides and be useful in cardiovascular events risk stratification regarding HF. Assessing many HF markers simultaneously may enhance better management and prognosis in HF patients. Higher CTS levels may help clinicians better manage and more aggressively treat these patients. Yet, such findings are still based on limited data. Aside from CTS serving as a diagnostic or prognostic tool, it also shows encouraging potency as an innovative therapeutic agent in many pathological conditions associated with chronic inflammation, including autoimmune, metabolic disorders, and CVDs [13]. The fact should be further thoroughly investigated as some preclinical documentation has already demonstrated CTS’s positive cardioprotective and hemodynamic effects [8,14]. Nonetheless, more large-scale studies are needed to confirm these findings. In this review, the authors aim to discuss the role of CTS in the development of different CVDs and to determine whether this molecule could serve as a prognostic biomarker as well as a potential therapeutic target for these diseases in the future.

The authors searched for relevant manuscripts in scientific databases (Google Scholar and PubMed) using the following search queries: “catestatin” AND “cardiovascular disease”; “catestatin” AND “arterial hypertension”; “catestatin” AND “coronary artery disease”; “catestatin” AND “heart failure”.

## 2. Catestatin in the Regulation of Blood Pressure

HA is one of the most prevalent cardiovascular diseases worldwide. While it is the most common modifiable risk factor associated with cardiovascular morbidity and mortality, many patients still do not achieve recommended blood pressure (BP) targets. This occurs even though proven approaches to lower BP, like diet modification and pharmacotherapy, are widely available [15]. It is, then, crucial to implement novel, adequate strategies for HA management.

**Figure 1:**
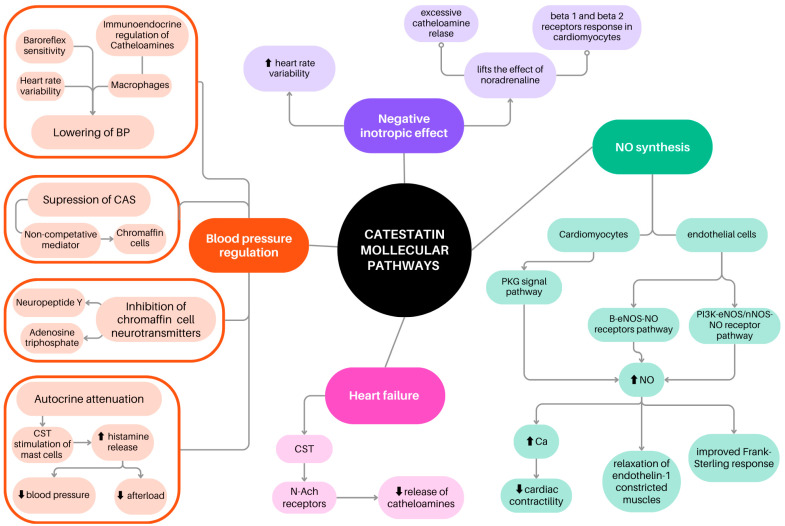
Catestatin molecular pathways [7,17] and involvement in cardiovascular diseases: blood pressure regulation [7,8,18,19,20,21], inotropic effect [22,23], and heart failure [24].

CTS has been established as a pleiotropic peptide that regulates the cardiovascular system, inflammatory processes, autoimmune reactions, and metabolic homeostasis [14]. Previous research on this molecule has suggested that CTS may contribute to the pathogenesis of HA. The mechanisms that have already emerged for cardiovascular effects of CTS are autocrine inhibition of catecholamine (CA) secretion from adrenal medullary chromaffin cells and adrenergic neurons, paracrine stimulation of histamine release from mast cells, and modulation of sympathetic and parasympathetic activities by acting at the baroreceptor center of the nucleus tractus solitarius [16]. CTS has been reported to suppress the release of CAs by acting as a noncompetitive mediator of the nicotinic cholinergic stimulation of chromaffin cells and by adenylate cyclase-activating polypeptide stimulation. CTS not only limits CA secretion but also inhibits the release of other chromaffin cell neurotransmitters, such as neuropeptide Y and adenosine triphosphate [7]. CTS may reduce BP, regulating baroreflex sensitivity and heart rate variability [8]. CTS involvement in various molecular pathways is presented in Figure 1.

Ying et al., in their study, generated mice with knockout (KO) of the region of the *CgA* gene that only coded CTS (CTS-KO). The CTS-KO mice were hypertensive. The administration of exogenous CTS rescued their high BP, whereas CTS did not alter mice with normal BP. Furthermore, they demonstrated that a raised abundance of macrophage infiltrates in the adrenal medulla coexisting with increased levels of CAs leads to the subsequent development of HA in CTS-KO mice. These results may suggest that CTS regulates the BP by influencing the immunoendocrine regulation of CA secretion via macrophages, which may play a key role as effector cells for the antihypertensive activity of CTS and, alongside the chromaffin cells, be the primary source of circulating CTS. Elevated BP in CTS-KO mice is implied to result from a lack of CTS, known as the endogenous inhibitor of nicotine-evoked catecholamine ejection [18]. Previous research has shown the association between nicotine-induced elevated BP and increased CA secretion [19]. Ying et al. proved it using chlorisondamine, which lowered BP in CTS-KO mice [18]. Hence, CTS is assumed to be the autocrine attenuator of cardiac inflammation in HA. The most important recent animal and in vitro studies on CTS are summarized in Table 1.

Jianqiang et al. explored wavering levels of plasma CTS in Spontaneously Hypertensive Rats (SHRs) and their littermates, Wistar–Kyoto rats (WKYs), and found that they were significantly higher in SHRs than in WKYs. Along with HA development and progression in SHR, plasma CTS levels gradually rose. The reduction in the heart rate of SHRs after exogenous administration of CTS may prove that it inhibits sympathetic activity in hypertensive individuals [20]. CTS acted as a potent vasodilator, and this effect was mediated by an elevated release of histamine. Kruger et al. showed that the most likely mechanism by which CTS might lead to histamine release would be stimulating mast cells [21]. Considering the central nervous system (CNS), CTS is reported to exert both sympathoexcitatory and cholinergic effects [8]. Administering CTS into the rat rostral ventrolateral medulla (RVLM), which is responsible for BP control in the brain stem, led to sympathoexcitatory effects, increased barosensitivity, and depletion of chemosensitivity and the somatosympathetic reflex, resulting in high BP [32]. On the contrary, the injection of CTS into the rat caudal ventrolateral medulla (CVLM) contributed to decreased barosensitivity and the depletion of the peripheral chemoreflex, resulting in low BP [33]. Furthermore, the injection of CTS into the rat’s central amygdala also decreased BP providing protection against vascular dementia and neurodegeneration [34]. These reports indicate that CTS plays a significant role in cardiorespiratory control in the CNS. Reduced plasma CTS levels and increased CgA levels have been noted in humans with HA [35]. O’Connor et al., in their study, demonstrated decreased plasma levels of CTS in normotensive offspring with a family history of HA. Dysregulation in the processing of CgA to CTS and CTS lowering may appear in the early stages of HA development, even though categorization into normotensive and hypertensive groups did not reveal significant differences in plasma CTS levels [35].

The most important recent human studies on CTS are summarized in Table 2. Durakoglugil et al. found that the difference in CTS levels between untreated patients with HA and healthy individuals was irrelevant after adjusting for age, gender, height, and weight [36]. Meng et al. explored levels of plasma CTS in HA patients and the relationship between CTS and left ventricular hypertrophy (LVH). Their results showed elevated CTS plasma levels in the HA group and lower CTS to norepinephrine ratio in patients with LVH compared to normal controls, suggesting that this molecule might participate in developing HA and LVH [37]. In contrast, in another study by O’Connor et al., CTS was significantly reduced in patients suffering from HA [38]. Choi et al. identified CTS genotypes and performed a genotype–phenotype association analysis on 343 participants from the Japanese population. The results showed that BP was higher in Gly/Ser subjects than in the wild-type Gly/Gly individuals. Accordingly, it was demonstrated that the CTS variant allele Ser-364 may be associated with higher BP in the Japanese population [39]. On the contrary, the Ser-364 allele was linked with lowered diastolic BP levels only in males in a Southern California study population. It seemed to reduce the risk of developing HA in men as well. The effect was not observed for SBP or in women [40].

Furthermore, due to its neuroprotective potential, CTS might be a novel target for the treatment and prevention of HA [34]. However, the results of the aforementioned studies are inconsistent, and it is too soon to draw conclusions.

## 3. Inotropic Effect of Catestatin and Association Between Catestatin and Heart Rate Variability

CTS has a negative inotropic effect, improves cardiomyocyte condition after episodes of ischemic reperfusion, and lengthens cardiomyocyte survival rate. CTS protects the heart and bloodstream from overdrive caused by the excessive release of catecholamines, such as norepinephrine and epinephrine. Furthermore, CTS lifts the effects of noradrenalin on Beta1 and Beta2 receptors in cardiomyocytes. Moreover, it prevents excessive myocardial remodeling and reduces the formation of reactive oxygen species [22].

Higher heart rate variability (HRV) values are commonly found during a relaxed state. However, people with a high HRV may have better stress resilience or better cardiovascular function [54]. Researchers performed several studies to explore the influence of CTS on the HRV. In patients with acutely decompensated HF, a higher level of CTS meant a lower heart rate [55]. A different study demonstrated that CTS regulates and prevents tachycardia in mice. In that study, researchers supplemented CgAknockout mice with CTS, which protected the subjects from high systolic blood pressure and decreased HRV [23]. Confirmation of these reports is provided by Dev et al. They performed an experiment in which supplementation of CTS increased HRV [56].

## 4. Catestatin İnfluence on NO Synthesis and Metabolism

CTS increases NO synthesis both from cardiomyocytes and endothelial cells. NO is produced in the NO(NOS)-NO-cGMP-cGMP-protein kinase (PKG) signal pathway and released as a result of the endothelial receptor B-eNOS-NO or PI3K-eNOS/nNOS-NO pathway [7,17]. Released NO reduces cellular Ca2+, which results in decreased cardiac contractility and negative lusitropic and inotropic effects. Moreover, NO causes the relaxation of endothelin-1 preconstricted coronaries and smooth muscles in the endothelium of other vessels. CTS increases the rate of positive Frank–Starling responses by increasing NO production. Because of that, it is suggested that CTS is a NO-dependent modulator of the regulation of the heart [7,17]. This correlation between NO synthesis and its impact on the cardiovascular system is crucial based on the results of several recent studies on CTS and its antiadrenergic effect. The antiadrenergic effects of CTS on the heart depend not only on a direct impact on papillary muscle cells but also on an increased rate of NO synthesis by endothelial cells [17]. Using inhibitors for specific points of the NO synthesis pathway in cardiomyocytes eliminates the negative inotropic effect exhibited by CTS [17]. Furthermore, using a selective eNOS inhibitor abolished the effect of CTS, and a neuronal NOS inhibitor only reduced this effect, suggesting that the effect of CTS on cardiac contractility is mediated primarily through the eNOS pathway.

Kiranmayi et al. determined a significant connection between the Ser-364 allele and HA in Chennai and the Chandigarh populations. They demonstrated that the Ser-364 individuals have higher BP levels. The CTS Ser-364 peptide, via altered interactions with ADRB2 and differential activation of extracellular regulated kinase (ERK) and endothelial nitric oxide synthase (eNOS) phosphorylation, led to greater modulation of the endothelial NO pathway compared with the CTS-WT peptide. This may contribute to the increased risk of HA in Ser-364 allele carriers, possibly due to lower NO production, which leads to diminished endothelial vasodilation. In conclusion, the Ser-364 allele appears to play an important role in HA development in Indian populations [57]. Another study on an Indian population found no association of this SNP with BP [58]. The Ser-364 allele seems to exert comparable effects on BP in different Asian populations but not in Caucasians. These contradictory associations determine heterogeneity in different populations. Thus, this proves the need to conduct more studies on diverse ethnic populations. Further studies concerning the underlying mechanisms of action of this physiological antihypertensive peptide may bring significant insights into the pathogenesis of cardiovascular diseases.

A study using cardiomyocytes isolated from rat papillary muscles and endothelial cells obtained from buffalo aortas showed, in even more detail, that CTS affects myocardial contractility mainly through NO synthesis. They used inhibitors of individual points of the NO synthesis pathway (PI3K, NOS) in the presence of CTS, which abolished its antiadrenergic effect. The endocardial endothelium layer was also removed and treated with CTS, which did not abolish the inotropic effect induced by the β adrenoceptor agonist- isoproterenol under basal conditions but completely abolished the antiadrenergic effect induced by wild-type CTS [59].

## 5. Catestatin in Coronary Artery Disease and Atherosclerosis Development

Despite significant advances in the diagnosis and treatment of coronary artery disease (CAD), it remains a leading cause of mortality and Disability Adjusted Life Years (DALYs) worldwide, resulting in 7 million deaths and loss of 129 million DALYs each year, which places CAD among the most serious threats to global health [60].

Atherosclerosis is the primary and most widely studied risk factor for CVDs, including CAD. It is an intravascular chronic inflammation process initiated by malfunction and increased endothelium permeability in the luminal layer of arteries or the intima. An elevated concentration of low-density lipoproteins (LDLs) in the serum promotes their accumulation in the intimal subendothelial space, followed by biochemical modifications, including oxidation, acetylation, glycosylation, glycoxidation, carbamylation, and others, primarily related to exposure to reactive oxygen species [61]. Modified LDLs in the presence of other atherogenic factors, such as nicotine addiction, DM, turbulent blood flow, and HA, lead to endothelial cell activation and monocyte recruitment in the intima [62,63]. LDLs are phagocyted by macrophages and accumulate in vascular smooth muscle cells, which migrate from the tunica media, and macrophages, forming foam cells and fatty streaks [64]. Over time, apoptosis is impaired, and cell necrosis predominates. A necrotic core, cell-impoverished, rich-in-lipids region characterized by an inflammatory microenvironment is formed. A fibrous cap protects the arterial lumen from the necrotic core’s prothrombotic properties. At this point, atherosclerotic plaque is well-developed and unlikely to disappear. Further expansion of the necrotic core results in arterial lumen reduction and blood flow disorders, rising atherosclerotic plaque instability, and the possibility of plaque rupture, eventually causing potentially lethal complications such as myocardial infarction (MI), ischemic stroke, and peripheral arterial disease [65].

The impact of CTS on atherosclerosis and CAD development and course is multifaceted and, despite the great interest of scientists in the last years, not yet completely clarified. It has been proven that CTS modulates the immune system, suppresses tissue inflammation, reduces reactive oxygen species production, attenuates stimulatory effects of catecholamines, reduces oxidative stress-induced cardiomyocyte apoptosis, and plays an essential role in monocyte migration and differentiation [22,29,66]. In addition, CTS has a beneficial influence on endothelial NO synthesis, glycemia, serum lipids levels, and arterial blood pressure, which has been extensively described in other sections of this article.

Although CTS by itself exhibits weak chemotactic properties in vitro and in vivo in rodent models, it has been shown to block monocyte and neutrophil migration that is dependent on chemotactic factors such as CCL2, CXCL2, and IL-8 [28].

Moreover, CTS-KO mice were characterized by increased serum levels of proinflammatory cytokines, including TNF-α, IFN-γ, CCL2, CCL3, and CXCL-1, whereas the anti-inflammatory IL-10 concentration was decreased. Furthermore, CTS-KO mouse cardiomyocytes had a lower expression of anti-inflammatory genes for IL10, IL4, Mrc1, Arg1, Clec7a, and Clec10a and upregulated proinflammatory genes, such as Tnfa, Ifng, Emr1, Itgam, Itgax, Nos2a, IL12b CcL2, and CxcL1. Those effects were fully reversible by exogenous CTS supply. Additionally, intensified phosphorylation (Ser177/181) of part of IKK-β, the inflammatory NF-κB signaling pathway, was observed in CTS-KO mice [18].

### 5.1. Catestatin and Angiogenesis After Myocardial İnfarction

Lener et al. showed that CTS stimulates the proliferation of human coronary artery smooth muscle cells and endothelial cells in Matrigel assays. The capillary-like tube formation stimulated by CTS was comparable to the effect acquired after administering the vascular endothelial growth factor (VEGF). Furthermore, CTS demonstrated antiapoptotic potential for human cardiomyocytes in vitro [26].

Moreover, the association between higher serum CTS levels and better collateral development in patients with chronic total occlusion of the coronary artery (CTO) has been reported. A total of 38 CTO patients and 38 healthy individuals were included in the study. The plasma CTS level was lower in the control group than in the CTO group (1.36 ± 0.97 ng/mL vs. 1.97 ± 1.01 *p* = 0.009). Within the CTO group, patients with good collateral development had higher CTS and VEGF levels than patients with unsatisfactory collateral development (2.36 ± 0.73 vs. 1.61 ± 1.12 ng/mL, *p* = 0.018; 425.23 ± 140.10 vs. 238.48 ± 101.00 pg/mL, *p* < 0.001) [67].

### 5.2. Catestatin Antiarrhythmic Potential

The occurrence of ventricular tachycardia (VT) or ventricular fibrillation (VF) is a common and serious threat to patients after MI, especially in an early phase of ischemia. Ventricular arrhythmias can be a direct cause of sudden cardiac death (SCD) and worsen short- and long-term prognosis for patients in general [68].

Zhang et al. described an experiment in a rodent model in which the left anterior descending coronary artery was ligated and electrically stimulated, resulting in ventricular tachyarrhythmia. The rodents were randomly divided into the control and the CTS administration groups. The patch-clamp technique was used to monitor the action potential, transient outward potassium current, delayed rectifier potassium current, inward rectifying potassium current, and L-type calcium current in rodent cardiomyocytes. Intensified Ito, IK, and IK1 activity was observed in the CTS group. Simultaneously, CTS inhibited ICa-L, shortening action potential time and reducing ventricular arrhythmia [25]. However, Pei et al. showed that a higher CTS level was associated with increased malignant arrhythmia incidence during hospitalization in patients with acute MI [69].

### 5.3. Catestatin as a Potential Coronary Artery Disease Course Marker

Xu et al. measured plasma CTS levels on admission in 170 patients with a suspected acute coronary syndrome (ACS) who had an emergency coronary angiography, of which 46 had MI with ST-segment elevation (STEMI), 89 had unstable angina pectoris (UAP), and 35 did not have CAD. The patients were followed up for two years to check if there would be any major adverse cardiovascular events (MACEs), such as recurrent acute MI, rehospitalization for HF, revascularization, and death due to cardiovascular causes. The plasma CTS level was higher in individuals without CAD (1.38 ± 0.98 ng/mL; *p* = 0.001) than in the STEMI (0.80 ± 0.62 ng/mL) and UAP (0.99 ± 0.63 ng/mL) groups. In multivariable linear regression analysis, body mass index, the presence of HA, and the type of CAD were independently associated with plasma CTS levels. There was no significant difference in the occurrence of MACEs between high and low CTS levels groups [42].

To explore this issue further, Xu et al. followed up on 165 patients with acute MI for four years. At the beginning of the study, the plasma CTS level and relevant medical data were collected. During the observation time, 24 patients had MACEs. The group experiencing MACEs exhibited notably lower plasma CTS levels (0.74 ± 0.49 ng/mL compared to 1.10 ± 0.79 ng/mL, *p* = 0.033) and had a higher average age (59.0 ± 11.4 years vs. 53.2 ± 12.8 years, *p* = 0.036). The incidence of MACEs was significantly greater in the older population (aged 60 and above) compared to the younger group (under 60 years old) (23.8% [15 out of 63] vs. 8.8% [9 out of 102], *p* = 0.008). Additionally, CTS levels were significantly lower in the MACEs group than in those without MACEs (0.76 ± 0.50 ng/mL vs. 1.31 ± 0.77 ng/mL, *p* = 0.012). Among older people, CTS levels were significantly linked to MACEs (Kaplan Meier, *p* = 0.007), whereas this association was not observed in the younger group (Kaplan Meier, *p* = 0.893). In the Cox proportional hazards regression analysis, elevated CTS levels emerged as an independent predictor of MACEs after controlling for other risk factors (hazard ratio [HR] = 0.19, 95% confidence interval [95% CI] = 0.06–0.62, *p* = 0.006) in older patients [41].

Chen et al. compared CTS serum concentrations between 224 patients with CAD and 204 healthy control group members in multistage research. The CTS serum level was lower in CAD patients than in healthy individuals [1.14 (1.05–1.24) ng/mL vs. 2.15 (1.92–2.39) ng/mL, *p* < 0.001]. A correlation between CTS level and CAD severity among 921 CAD patients was studied in the subsequent step. It turned out to be inversely correlated with CAD severity (r = −0.208, *p* < 0.001) [30]. On the other hand, higher serum CTS levels in CAD patients were reported.

Furthermore, some studies showed an association between higher serum CTS levels and worse prognosis. Zhu et al. followed up on 72 patients with STEMI for 65 months. CTS serum levels were measured at admission and on day 3 and day 7 after STEMI. Echocardiographic parameters were evaluated on day 3 and at the follow-up’s end. Patients with higher serum CTS levels at day 3 had worse left ventricular function and a higher risk of left ventricular remodeling [46]. Zhu et al. evaluated 100 patients after MI and successful percutaneous coronary intervention. The patients were followed up for 65 months for endpoints such as death from cardiovascular causes, readmission with the ACS, or admission with congestive HF. Negative endpoints were associated with higher CTS levels after admission. Liu et al. followed up on 120 patients with coronary heart disease for 1045 days. No association between CTS levels on admission and MACEs was observed in this study [43].

Scientific reports on CTS levels in patients with CAD are inconclusive. Both increased and decreased levels of CTS have been reported as a positive prognostic factor in CAD patients. The studies’ results might be puzzling due to differences in the study group selection and different endpoints reported. Multicenter prospective clinical trials in this area could address this issue.

## 6. Catestatin in Heart Failure

HF is an ineffective ability of the heart to pump blood efficiently through the body. According to a recent report from 2024, the number of Americans over the age of 20 who suffer from diseases of the heart, including HF, is estimated to be around 127.9 million, which is nearly 50% of the country’s total population. However, in 2019, a much lower number was suggested globally. Across 204 countries, the number of people living with HF was around 56.2 million [70]. Neurohormonal changes aim to optimize the cardiac function of patients with HF. The activation of the renin–angiotensin–aldosterone axis and the sympathetic nervous system at first allows the body to manage impaired cardiac output by optimizing preload and afterload levels. However, over time, the process is counterproductive due to excessive peripheral vascular resistance.

Many researchers have discovered that CTS is an important member of a group of substances regulating homeostasis in the human organism [47,53,71]. CTS is stored and released, along with catecholamines, in the neuronal endings of the sympathetic nervous system. CTS affects N-Ach receptors, which decrease the release of catecholamines. Additionally, CTS causes the release of histamine, which directly causes vasodilatation. These processes lower blood pressure and, in turn, reduce the afterload. A lower afterload decreases the severity of HF [24].

A study showed that higher levels of CTS were noted in patients who died than in patients who survived, and the authors concluded that CTS plays an important protective role in managing cardiac output in HF patients. Additionally, patients suffering from HF with ischemic origin present higher concentrations of CTS compared to non-ischemic HF [45]. A higher concentration of CTS was found in patients in the acute phase of decompensated HF of ischemic etiology. In light of this information, CTS can be viewed as a marker of sympathetic activation [72]. Moreover, researchers concluded that CTS could be a better, more sensitive marker for patients with HF whose ejection fraction was mildly reduced and preserved than patients with a reduced fraction [44]. It was confirmed that the higher the level of CTS, the more likely CV death may occur [24,44]. Researchers performed tests on animal models and proved that CTS has a protective influence on cardiomyocytes in mice with HFpEF. In this study, injections with CTS protected against the decline of ejection fraction and remodeling of cardiomyocytes [24].

In one study, the perfusion rate and left ventricular pressure (LVP) were monitored in rat hearts to further understand CTS’s role in the process. In isolated cardiomyocytes, CTS improved the survival rates of cardiomyocytes by 65% after a simulated HF [73]. Furthermore, a similar treatment in rats with HF after MI gave promising results. CTS caused better capture of Ca2+ by atriums and lowered the probability of atrial fibrillation [74].

However, an elevated CTS level may correspond to acute worsening of HF with ischemic etiology and is most likely a way of compensating for increased sympathetic activity caused by the disease [45]. Another piece of research confirmed that the concentration of CTS is higher in HFrEF with a higher NYHA class, and correlates with NT-proBNP. The authors estimate that CTS could be a prognosis marker in HF because a higher level of CTS was linked with the worse long-term state of patients and all-cause death [11].

## 7. Catestatin in Other Diseases

### 7.1. Catestatin in Pre-Eclampsia

Pre-eclampsia is a serious and potentially lethal condition that may occur during pregnancy. With its significant impact on mortality and morbidity of mothers and newborns, it is a serious threat to global health. In women who have experienced pre-eclampsia, increased risks of metabolic and CVDs, such as DM, stroke, and MI, are observed, which results in reduced life expectancy. Moreover, children from pregnancies with pre-eclampsia are at increased risk of preterm birth and perinatal death. They are also in danger of neurodevelopment disability, metabolic issues, and CVDs in the future. Pre-eclampsia is a multiple-systems disease characterized by suddenly emerging HA after the 20th week of pregnancy and at least one of the following conditions: proteinuria, uteroplacental, or another organ dysfunction. A dysfunctional placenta releases a variety of proinflammatory cytokines into the circulatory system, which results in systemic inflammation, endothelial dysfunction, and, finally, the occurrence of seizures—eclampsia. Pre-eclampsia can be treated symptomatically with hypotensive drugs and anticonvulsants, which are not indifferent to the child. However, it can be cured only by termination of pregnancy, in many cases prematurely, and removal of the defective placenta [75]. The production and secretion of CgA and, indirectly, CTS occur in placental cells. CTS regulates vascular blood pressure and angiogenesis in the placenta as well as the proliferation, migration, and apoptosis of placental cells [76].

Bralewska et al. tested the expression of the CgA gene and the CTS levels in placentas among 102 patients with pre-eclampsia and 103 healthy pregnant women as a control group. Although the expression of placental CgA was significantly higher in the pre-eclampsia group (−0.25 ± 1.7 vs. −0.82 ± 1.5, *p* = 0.011), the mean CTS level in placentas in the pre-eclampsia group was lower than in the control group (6.4 ± 1.0 vs. 6.7 ± 1.4, *p* = 0.04) [49]. A study on HTR-8/SVneo and BeWo Trophoblastic Cell Lines showed that CTS acts as an antiapoptotic factor. As a blood pressure-lowering agent, it also acts as a compensatory mechanism. It appears that CTS deficiency in trophoblasts may favor the occurrence of pre-eclampsia [27].

Tüten et al. compared serum CTS levels among 50 pregnant women with mild pre-eclampsia, 50 pregnant women with severe pre-eclampsia, and 100 healthy women with uncomplicated pregnancy and matched gestational age as a control group. Mean serum CTS was significantly higher in the pre-eclampsia group compared to the control group (290.7 ± 95.5 pg/mL vs. 182.8 ± 72.0 pg/mL; *p* < 0.001). Mean serum CTS was not significantly different in mild and severe pre-eclampsia groups (282.7 ± 97.9 pg/mL vs. 298.7 ± 93.4 pg/mL, *p* = 0.431) [48].

On the other hand, Palmrich et al. reported that CTS serum levels among 50 women with pre-eclamptic pregnancies were lower than in 50 healthy controls with matched gestational age (median CTS: 3.03 ng/mL, IQR [1.24–7.21 ng/mL] vs. 4.82 ng/mL, IQR [1.82–10.02 ng/mL]; *p* = 0.01). The difference in CTS levels between early- and late-onset pre-eclampsia groups was not statistically significant.

Özalp et al. also compared maternal serum CTS levels in 27 early-onset pre-eclampsia individuals and 28 late-onset pre-eclampsia with a control group of 28 healthy pregnant women. However, the differences between the groups were not statistically significant. Interestingly, researchers found correlations between maternal serum CTS levels and fetal echocardiographic parameters: The fetal E/A ratio was positively correlated with maternal CTS levels (*p* < 0.001) in both the pre-eclampsia and control groups. Fetal isovolumetric relaxation time and fetal myocardial performance index values were negatively correlated with CTS levels in both the pre-eclampsia (*p* < 0.001, *p* = 0.001, respectively) and control group (*p* < 0.001, *p* = 0.002, respectively) [50].

CTS plays an important, but not fully understood, role in the pathogenesis of pre-eclampsia. Early diagnosis and initiation of treatment are crucial to ensuring the safety of mother and child, which is often possible only in highly referential obstetric clinics. CTS appears to be a promising marker in this disease as well as other maternal and fetal CVDs. However, scientific reports in this area are not conclusive, and further research is necessary.

### 7.2. Catestatin in Acute Pulmonary Embolism

Acute pulmonary embolism is a dangerous condition characterized by the narrowing or occlusion of a pulmonary artery or its branches by embolic material. It is most often formed by thromboses in the deep veins of the lower extremities. However, in rare cases, embolism from fat, amniotic fluid, neoplasm cells, or air may be the cause. To estimate the patient’s risk of death within 30 days of the onset of acute pulmonary embolism, the validated Pulmonary Embolism Severity Index (PESI) score can be used. Depending on the severity of the disease and the capacity of the medical center, it can be treated pharmacologically by administering anticoagulants or invasive mechanical embolectomy [77,78].

It was demonstrated, in a rodent model, that CTS has an important protective role in acute pulmonary embolism. Eight-week-old C57/BL6 mice were divided into the following groups: the control group with no CTS treatment, the control group with CTS treatment, the induced acute pulmonary embolism with CTS pre-treatment group, and the induced acute pulmonary embolism without CTS pre-treatment group. Pulmonary embolism was induced by administration of collagen and epinephrine. CTS levels were decreased, while platelet numbers, von Willebrand factor, E-selectin, P-selectin, myeloperoxidase, and monocyte chemoattractant protein 1 serum levels were increased in the pulmonary embolism group compared to the control group. Interestingly, this growth was less intense in the group after CTS pre-treatment. Platelet numbers were negatively correlated with CTS levels (r = 0.6732, *p* = 0.002). Survival rates 30 min after acute pulmonary embolism onset were 30% in the group without CTS pre-treatment and 80% in the group with CTS pre-treatment, respectively. Lung samples were collected and assessed in histopathological examination. In acute pulmonary embolism groups, pulmonary embolization, damaged walls of alveoli, and immune cell infiltration were observed. However, pathological changes in the group after CTS pre-treatment were less severe. In mouse models, CTS mitigates endothelial inflammation and promotes thrombus resolution in acute pulmonary embolism. According to the authors, the beneficial effects of CTS are multi-causal and include inhibition of endothelial inflammation by the TLR-4-p38 signaling pathway [31].

On the other hand, Izci et al. analyzed 84 men and 76 women with acute pulmonary embolism. The sPESI scores were calculated for all patients. CTS levels in plasma among patients with acute pulmonary embolism were higher than in healthy individuals (17.5 ± 6.1 ng/mL vs. 27.3 ± 5.7 ng/mL, *p* < 0.001). In a group with sPESI ≥ 1, plasma CTS levels were higher than in a group with sPESI < 1 (37.3 ± 6.1 vs. 24.2 ± 5.3 ng/mL, *p* < 0.001). Furthermore, a positive correlation was found between CTS plasma level and sPESI score (*p* < 0.001). During the hospitalization, 20 deaths in the group with sPESI ≥ 1 (mortality 27.7%) and 9 deaths in the group with sPESI < 1 (mortality 10.2%) were reported (*p* = 0.01). Receiver operating characteristic (ROC) curve analysis with a cut-off CTS serum level of 31.2 ng/mL was performed. It showed that CTS level predicted mortality with 100% sensitivity and 52.6% specificity (AUC = 0.883, 95% CI: 0.689–0.921). CTS level was correlated with dysfunction of the right ventricle [52].

Based on available publications, CTS positively affects the course of acute pulmonary embolism in an animal model and in vitro and may be a promising disease course marker. However, the available data on CTS levels during acute pulmonary embolism are inconclusive. The differences that occurred might be related to the methods of measurement, the time between disease onset and sample collection, or differences in CTS metabolism between humans and rodents. There are limited data available in this area and further research is necessary.

### 7.3. Catestatin in Chronic Kidney Disease

Chronic kidney disease (CKD) is characterized by progressive loss of renal function. Sodium retention, volume expansion, inflammation, oxidative stress, overactivity of the sympathetic nervous system, mineral bone disorders, hormonal disorders, and uremic toxins play important roles in CKD progression and cardiovascular complications occurrence. Generalized atherosclerosis with a significant tendency to intense calcification is typical for CKD. Patients with CKD have an increased risk of HF, arrhythmias, CAD, or SCD. CVDs remain the first cause of death in G5 CKD patients on hemodialysis, with 20 times higher CVD-related mortality than in the general population. Furthermore, many patients with CKD die due to CVD complications before reaching end-stage renal dysfunction [79,80].

Luketin et al. compared plasma CTS levels and advanced glycation end products (AGEs) among 91 hemodialysis patients and 70 healthy individuals. AGEs promote atherosclerosis in an inflammatory environment, leading to cardiovascular events among patients. The study showed that plasma CTS levels were increased in the hemodialysis group compared to the control group (32.85 ± 20.18 vs. 5.39 ± 1.24 ng/mL, *p* < 0.001). The CTS serum level was positively correlated with AGE levels (r = 0.492, *p* < 0.001). Moreover, CTS serum levels were also positively correlated with both the Dialysis Malnutrition Score (r = 0.295, *p* = 0.004) and the Malnutrition-Inflammation Score (r = 0.290, *p* = 0.005) [51].

Sun et al. followed up on 330 hemodialysis patients for 36 months. In that period, 29 deaths due to CVD and 28 deaths due to other diseases were reported, and one patient was lost. The multivariate regression analysis among 272 alive patients showed an association between plasma CTS level ≥ 1.9 ng/mL and increased risk of cardiac death (RR 6.13, 95% CI 2.54, 18.45). Furthermore, survival analysis showed an increased rate of cardiac death in the group with plasma CTS levels ≥ 1.9 ng/mL than in the group with plasma CTS levels < 1.9 ng/mL (*p* < 0.001). Also, the overhydration to total body weight ratio and daily diuresis were linearly correlated with plasma CTS level (r = 0.502, *p* < 0.001 and r = −0.338, *p* < 0.001) [79].

According to the available scientific data, increased CTS serum levels are typical for hemodialyzed patients and can be linked to a worse prognosis. It should be assumed that CTS plays an important role in the pathophysiology of cardiovascular complications in CKD hemodialysis patients, and it could be a promising novel marker of disease severity. However, the availability of scientific evidence is limited, and confirmation in further research is necessary.

## 8. Conclusions and Future Perspectives

To conclude, cardiovascular disease pathogenesis is an important and still not fully understood field of study. Studies on CTS are promising and show the possibility of using CTS as a treatment for many CVDs. Additionally, CTS increases the production of NO in cardiomyocytes and endothelial cells. CTS’s role in the development of CVDs and its potential as a therapeutic target are interesting topics in cardiology. The aforementioned studies suggest the importance of this peptide for the development of common diseases, which remain a serious and expensive problem for healthcare systems worldwide. Moreover, CTS can be a biochemical marker for patients with preserved and mildly reduced HF. On the other hand, there are diseases in which the role of CTS is still not fully understood, e.g., atherosclerosis—studies about the influence of CTS on the remodeling of the vessel walls are inconsistent. Some of the reported studies are just preliminary to the subsequent phases of research on CTS involvement in CVDs. However, it is too soon to generalize CTS’s importance for CAD, HF, and AH.

## Figures and Tables

**Table 1:** Most important recent animals and in vitro studies on catestatin.

Study and Its Reference	Methodology	Results Summary
Zhang et al. 2024 [25]	Fourty-two male adult Sprague–Dawley rats randomly divided into CTS and NON-CTS equinumerous groups. Ventricular arrhythmias were induced by ligation of the LAD and electrical stimulation.	**CTS notably reduced induced ventricular arrhythmia caused by ischaemia and electric stimulation in rats.** In the CTS group: **↑**Ito, **↑**IK, **↑**IK1, and **↓**ICa-L activity.
Lener et al. 2023 [26]	Matrigel assays; human coronary artery endothelial cells (HCAECs) and human coronary artery smooth muscle cells (HCASMCs)	**CTS induces chemotaxis of HCAECs** (relative CI CTS 1 nM 1.79 ± 0.1, n = 3, *p* < 0.01 vs. control) similar to VEGF (relative CI VEGF 50 ng/mL 2.13 ± 0.09, n = 3, *p* < 0.01 vs. control). **CTS stimulates HCAEC proliferation** (relative proliferation CTS 1 nM 1.62 ± 0.05, n = 5, *p* < 0.01). **CTS had similar effect on capillary tube formation as VEGF** (relative tube formation CTS 1 nM 2.42 ± 0.1, n = 3, *p* < 0.001 vs. control; relative tube formation VEGF 50 ng/mL 2.06 ± 0.1, n = 3, *p* < 0.001 vs. control). **CTS activated ERK 1/2 signaling pathway in HCAEC.** **CTS stimulates HCASMC proliferation** (relative proliferation CTS 10 nM 1.6 ± 0.09, n = 3, *p* < 0.001 vs. control, relative proliferation CTS 1 nM 1.27± 0.04, n = 3, *p* = n.s. control) **CTS stimulated ERK 1/2 signaling and activation of the PI3-kinase-Akt pathway in HCASMC.** **CTS reduced H_2_O_2_ induced apoptosis** (relative apoptosis CTS 1 nM 0.76 ± 0.05, *p* < 0.05 vs. control)
Qiu et al. 2023 [24]	SG: C57BL/six male mice with TAC/DOCA induced HFpEF G1: 28 days of CTS treatment, n = 8 G2: 28 days of placebo, n = 8 C57BL/six male mice after thoracotomy without TAC/DOCA G3: 28 days of CTS treatment, n = 8 G4: 28 days of placebo, n = 8 ECHO evaluation, conductance catheter pressure-volume analysis, microscopy and genetic analysis of cardiomyocytes 7 weeks after surgery.	**CTS Protects Diastolic Dysfunction in mice with HFpEF.** In the TAC/DOCA (G2) group, **↑**E/A velocity ratio; **↓**E wave deceleration time; **↑**E/e′ suggesting diastolic dysfunction. **↑**LV end-systolic pressure, **↑**LV chamber stiffness, **↑**LV concentric hypertrophy, **↑**Volume of cardiomyocytes. **Those effects were reduced in TAC/DOCA + CTS (G1) group.** **Mitochondrial ROS generation reduction in TAC/DOCA + CTS (G1) compared to G2).** **Restoration of mitochondrial respiratory chain by CTS treatment.**
Bralewska et al. 2023 [27]	HTR-8/SVneo (CRL-3271) and BeWO (CCL-98) trophoblast cell lines incubated in pre-eclamptic environment (hypoxia, pro-inflammatory, oxidative stress)	Throphoblast cells produce CgA and CTS. Pre-eclamptic environment promotes **↓**CHGA gene expression (*p* < 0.001); ↓CTS level in trophoblast; **↑**apoptosis. **There is negative correlation between CTS level and apoptotic index** for both HTR-8/SVneo (R = 0.4) and BeWo cells (R = 0.5). **CTS acts as antiapoptotic factor in vitro.**
Muntjewerff et al. 2022 [28]	Transwell migration assays on human blood monocytes and neutrophiles; aortic ring model from Cx3cr1+/gfp transgenic mice.	**CTS itself has a weak chemotactic effect on monocytes and neutrophiles, however it counteracts the chemoattraction** of leukocytes by inflammatory chemokines CCL2, CXCL2, and IL-8 **CTS promotes angiogenesis.**
Ying et al. 2021 [18]	TG: CTS-KO C57BL/6 male mice, n = 8 CG: CTS-WT C57BL/6 male mice, n = 8	In CTS-KO mice: **↑SBP, ↑DSB, ↑MAP**. **Pro-inflammatory: serum cytokines ↑TNF-α, ↑IFN-γ, ↑CCL2, ↑CCL3, ↑CXCL**, genes upregulation: Tnfa, Ifng, Emr1, Itgam, Itgax, Nos2a, IL12b CcL2, and CxcL1. **Anti-inflammatory: serum ↓IL-10**, genes downregulation: IL10, IL4, Mrc1, Arg1, Clec7a and Clec10a. **Adrenal and plasma ↑catecholamines. ↑Sympathetic nerve activity.** Those effects were reversed by exogenous CTS administration. IPC-induced cardioprotection impairment. ↑phosphorylation (Ser177/181) of IKK-β (inflammatory NF-κB signaling pathway). Macrophages production: ↓TNF-α, ↓CCL-2, ↓CCL-3, ↓CXCL-1, ↓IL-1β, ↑IL-10 after CTS administration. **Macrophages themselves produce CgA and CTS.**
Alam et al. 2020 [22]	H9c2 myoblasts stimulated for sarcomere reorganization by Troponin T antibodies and norepinephrine.	**CTS attenuates myoblasts hypertrophy and suppresses the generation of ROS induced by norepinephrine; however, CTS does not protect cells from apoptotic signalling induced by norepinephrine.**
Chu et al. 2020 [29]	8 weeks old male Sprague–Dawley rats TG: Langendorff global ischemia/reperfusion model; CG: healthy rats; primary culture of cardiomyocytes from neonatal rats.	Average LV infarct size was33.66 ± 3.61% in I/R group. **Posttreatment with CTS reduced infarct size** to 20.25 ± 3.23% (*p* = 0.011 vs. I/R group). ↓LDH in myocardium in I/R group than in CG (6843.5 ± 1136.0) U/g vs. (102,470.0 ± 1066.1)U/g, *p* < 0.001. **CTS intervention reduced the ↓LDH** (8994.4 ± 963.8)U/g vs. (6843.5 ± 1136.0)U/g, *p* < 0.001). **CTS post-treatment decreased oxidative-stress and reduced apoptosis of cardiomyocytes after I/R. CTS reduced apoptosis in cardiomyocytes culture induced by H_2_O_2_ through activating the β2 adrenergic receptor and PI3K/Akt pathway.**
Chen et al. 2019 [30]	Atherosclerosis model: 8 weeks old male ApoE-KO mice divided into groups: G1 (CG) PBS i.p. G2: CTS i.p. G3 CTS + DX600 (ACE2 inhibitor) i.p. Human aortic endothelial cells (HAECs) and human umbilical vein endothelial cells (HUVECs)	**CTS reduced TNF-α-induced expression of IL-6, MMP-2 and adhesion molecules** (ICAM-1, VCAM-1, and E-selectin) in HAECs. **CTS promoted expression and activity of ACE2 in HUVECs. CTS reduced adhesion events and increased the rolling velocity of leukocytes,** those effect were blocked by ACE2 inhibitor though. **Plaque area of the aorta was reduced in the CTS-treated mice** vs. controls.
Chen et al. 2019 [31]	Eight-week-old C57/BL6 mice randomly divided into control (n = 20), control CTS (n = 20), APE (n = 20), and APE CTS (n = 20) Human pulmonary artery endothelial cells (HPAECs). APE in mice was induced by injection of collagen and epinephrine through the inferior vena cava	**Plasma CTS lower in APE** than in CG (*p* < 0.01) Negative correlation between CTS and Platelets level (Pearson correlation test r = 0.6732). **Survival rate 30 min after APE onset was higher in APE CTS** than in APE group (80% vs. 30%, *p* < 0.01). **CTS had anti-thrombotic activity in APE mice.** CTS inhibited APE-induced release of inflammatory neutrophils and macrophages. **CTS blocked TLR-4 p38 phosphorylation in HPAECs.**

APE—acute pulmonary embolism; CCL—C-C motif chemokine ligand; CG—control group; CgA—chromogranin A; CTS—catestatin; CXCL—C-X-C motif chemokine ligand; DSB—diastolic blood pressure; DOCA—deoxycorticosterone acetate; G—group; HR—hear rate; ICa-L—L-type calcium current; IFN-γ—Interferon gamma; IKK-β—inhibitor of nuclear factor kappa-B kinase subunit beta; IK—delayed rectifier potassium current; IK1—inward rectifier current; IL—Interleukin; IPC—Ischemic pre-conditioning; I/R—ischaemia/reperfuson; Ito—transient outward potassium current; KO—knockout; LAD—left anterior descending coronary artery; MAP—mean arterial pressure; ns.—not statistically significant; SBP—systolic blood pleasure; SHR—Spontaneously Hypertensive Rats; TAC—transverse aortic constriction; TG—test group; TNF-α—tumor necrosis factor alpha; VEGF—vascular endothelial growth factor; WKY—Wistar-Kyoto rats; WT—wild type.

**Table 2:** Most important recent clinical trials on catestatin.

Study and Its Reference	Methodology	Results Summary
**Coronary artery disease**		
Xu et al. 2022 [41]	Cohort study among 165 patients with AMI; 4 years follow-up for MACEs MACEs group n = 24. Young = age <60 years old Elderly = age ≥60 years old	**Lower CTS level in MACEs group** (0.74 ± 0.49 ng/mL vs. 1.10 ± 0.79 ng/mL, *p* = 0.033); MACEs rate was higher in the elderly group than in the young group (23.8% [15/63] vs. 8.8% [9/102], *p* = 0.008). **CTS level was lower in the MACEs group than in the non-MACEs group** (0.76 ± 0.50 ng/mL vs. 1.31 ± 0.77 ng/mL, *p* = 0.012) and **CTS was associated with MACEs (Kaplan Meier, *p* = 0.007) among the elderly group, but not in the young group** (Kaplan Meier, *p*= 0.893). **In the Cox proportional hazards regression CTS was independent factor for MACEs in elderly patients** (hazard ratio 0.19, 95% confidence interval 0.06–0.62, *p* = 0.006).
Chen et al. 2019 [30]	Cross-sectional study Stage 1 TG: 224 patients with CAD and CG: 204 healthy controls Stage 2 association between CTS and atherosclerosis severity in 921 CAD patients	**CTS lower in CAD patients than in CG** 1.14 (1.05–1.24) ng/mL vs. 2.15 (1.92–2.39) ng/mL, *p* < 0.001. **Negative correlation between CTS and atherosclerosis** severity (r = −0.208, *p* < 0.001)
Xu et al. 2017 [42]	Cohort study among 170 patients with suspected ACS who underwent coronarography TG: STEMI n = 46; UAP n = 89 CG: No CAD n = 35 2 years follow-up for MACEs	**Plasma CTS in STEMI group (0.80 ± 0.62 ng/mL) and UAP group (0.99 ± 0.63 ng/mL) were lower than in CG** (1.38 ± 0.98 ng/mL; *p* = 0.001). In multivariable linear regression, body mass index, presence of hypertension, and type of **CAD** were independently related to the plasma CTS level. However, there were no significant differences in MACEs between patients with high and low levels of CTS
Liu et al. 2013 [43]	Cohort study on 120 CAD and TG: SAP n = 15; UAP n = 47; NSTEMI n = 22; STEMI n = 36 CG: 30 healthy individuals CTS measurement at admission Median follow-up time: 1045 days	**CTS higher in TG than in CG** (1.02 ± 0.70 vs. 0.41 ± 0.14, *p* < 0.05) **CTS higher in SAP than in CG** (0.72 ± 0.50 vs. 0.41 ± 0.14, *p* < 0.05) **CTS higher in UAP than in CG** (0.88 ± 0.58 vs. 0.41 ± 0.14, *p* < 0.05) **CTS higher in NSTEMI than in CG** (1.05 ± 0.48 vs. 0.41 ± 0.14, *p* < 0.05) **CTS higher in STEMI than in CG** (1.31 ± 0.91 vs. 0.41 ± 0.14, *p* < 0.05) **CTS correlated positively with NE** (Spearman correlation coefficient r = 0.51, *p* = 0.00) **and NTproBNP** (r = 0.24, *p* = 0.01) **Plasma CTS on admission was not associated with adverse cardiovascular events.**
**Heart failure**		
Chu et al. 2024 [44]	A cohort study on 199 HF patients according to modified Framingham criteria. LVEF ≤ 40% n = 100; LVEF > 40% n = 102; Determination of CTS predictive value in HFrEF and HFmrEF/HFpEF respestively.	**Plasma CTS level had a moderate predictive ability for CV death** with a C statistic of 0.59 (95% CI 0.45–0.74), sensitivity 51.8%, specificity 71.8% in the HFrEF population and **better prognostic value** with C statistic of 0.72 (95% CI 0.59–0.85), sensitivity 70,8%, specificity 71,8% **in the HFmrEF/HFpEF population.**
Qiu et al. 2023 [24]	A cross-sectional study on 81 patients with HFpEF and 76 non–heart failure controls.	**Serum CTS level was higher in HFpEF group than in CG** (11.21 [interquartile range, 6.81–19.12] ng/mL vs. 23.62 [interquartile range, 11.53–34.81] ng/mL; *p* < 0.001). **Serum CTS level was positively correlated with NT-proBNP** level (r = 0.41; *p* < 0.001) and E/e′ ratio (r = 0.25; *p* = 0.002).
Borovac et al. 2020 [45]	Cohort study on 96 acute decompensated HF followed up until discharge Survivors n = 90 Non-survivors n = 6	**Serum CTS higher in non-survivors than in survivors** 19.8 (IQR 9.9–28.0) vs. 5.6 (IQR 3.4–9.8) ng/mL, *p* < 0.001. **CTS was an independent predictor of in-hospital death** (FC 6.58, 95% CI 1.66–21.78, *p* = 0.003). In ROC analysis CTS AUC (0.905 95% CI 0.792–1.000, *p* < 0.001).
Zhu et al. 2017 [46]	A cohort study on 72 patients with STEMI followed-up for 65 months and 30 healthy controls. Serum CTS measurement. ECHO.	**CTS levels correlated with the changes of LVEDD (*p* < 0.0001), EF (*p* = 0.0002), E (*p* = 0.0003), A (*p* < 0.0001), E’ (*p* < 0.0001), E/A (*p* < 0.0001), as well as E/E’ (*p* < 0.0001).**
**Pre-eclampsia**		
Palmrich et al. 2023 [47]	Cross-sectional study among 100 pregnant women. TG: 50 pre-eclamptic singleton pregnancy patients CG: 50 healthy pregnant women Serum CTS level comparison.	**CTS serum level in pre-eclamptic group lower than in CG** (median CTS: 3.03 ng/mL, IQR [1.24–7.21 ng/mL] vs. 4.82 ng/mL, IQR [1.82–10.02 ng/mL]; *p* = 0.010).
Tüten et al. 2022 [48]	Cross-sectional study among 2oo pregnant women. TG: 50 women with mild preeclampsia, 50 women with severe preeclampsia, CG: 100 healthy pregnant women	**Mean serum CTS increased in the preeclampsia group** than in CG (290.7 ± 95.5 pg/mL vs. 182.8 ± 72.0 pg/mL). No significant differences in CTS level between mild and severe preeclampsia groups (282.7 ± 97.9 pg/mL vs. 298.7 ± 93.4 pg/mL, *p* = 0.431). Serum CTS had positive correlations with systolic and diastolic blood pressure, urea, uric acid, and creatinine.
Bralewska et al. 2021 [49]	A cohort study of 205 pregnant women. TG: 102 pre-eclamtic patients CG: 103 healthy pregnant women Placental expression of the CgA gene and placental CTS level comparison.	**Placental expression of chromogranin A higher in pre-eclamptic patients** than in CG (−0.25 ± 1.7 vs. −0.82 ± 1.5, *p* = 0.011). **Mean CTS level lower in pre-eclamptic group** than in CG (6.4 ± 1.0 vs. 6.7 ± 1.4, *p* = 0.04).
Özalp et al. 2021 [50]	Cross-sectional study TG: 27 women with early-onset pre-eclampsia, 28 women with late-onset pre-eclampsia CG: 28 healthy pregnant women. Maternal serum CTS measurement and fetal ECHO.	**The fetal E/A ratio positively correlated with the maternal serum CTS levels in both the pre-eclampsia group and CG** (*p* < 0.001, *p* < 0.001). **Fetal isovolumetric relaxation time and MPI values negatively correlated with maternal CTS** in pre-eclampsia and CG (*p* < 0.001, *p* = 0.001, *p* < 0.001, and *p* = 0.002, respectively). **No significant CTS level difference between TG and CG.**
**Chronic kidney disease**		
Luketin et al. 2021 [51]	Cross-sectional study TG: 91 adult patients with end-term chronic kidney disease haemodialyzed > 1 year CG: 70 healthy adult individuals	**Plasma CTS higher in HD than CG** (32.85 ± 20.18 vs. 5.39 ± 1.24 ng/mL, *p* < 0.001). **Positive correlations between CTS and AGEs** (r = 0.492, *p* < 0.001) and **between CTS and both the Dialysis Malnutrition Score** (r = 0.295, *p* = 0.004) **and Malnutrition-Inflammation Score** (r = 0.290, *p* = 0.005).
**Pulmonary embolism**		
Izci et al. 2020 [52]	Prospectively study TG: 160 patients with contrasted CT-confirmed pulmonary embolism female n = 76; male n = 84 CTS measurement within 24 h after admission. CG: 97 healthy individuals female n = 55; male n = 42 Serum CTS measurement 0, 3 and 7 days after admission; ECHO	**Plasma CTS higher in APE group than in CG** (17.5 ± 6.1 ng/mL vs. 27.3 ± 5.7 ng/mL, *p* < 0.001). **Plasma CTS higher in the sPESI ≥ 1** (n = 72) than in the patients with sPESI < 1 (37.3 ± 6.1 vs. 24.2 ± 5.3 ng/mL, *p* < 0.001). **Positive correlation between CTS level and sPESI score** (±0.581, *p* < 0.001). ROC curve analysis with cut-off level of 31.2 ng/mL, and the **CTS level predicted mortality with a sensitivity of 100% and specificity of 52.6%** (AUC = 0.883, 95% CI: 0.689–0.921). **CTS level correlated with right ventricular dysfunction.** **Negative endpoints were associated with higher CTS levels after admission.**
**Rheumatoid arthritis**		
Pàmies et al. 2024 [53]	A cohort study of 199 rheumatoid arthritis patients. female n = 132; male n = 67	**RF-positive patients had higher CTS levels than RF-negative patients** (*p* < 0.001). Positive correlations between: CTS and LDL-C (ρ = 0.32, *p* = 0.009); CTS and IL-32 (ρ = 0.20, *p* = 0.003); CTS and fet-a (ρ = 0.20, *p* = 0.004). **↑CTS in women with DM2** (*p* = 0.04).

A—peak late diastolic mitral flow velocity; ACS—acute coronary syndrome; AGEs—advanced glycation end products; AMI—acute myocardial infarction; APE—acute pulmonary embolism; CG—control group; CTS—catestatin; DM2—type 2 diabetes mellitus; E—peak early diastolic mitral flow velocity; E’—doppler-derived peak early diastolic mitral flow velocity; ECHO—echocardiography; FC—firth coefficient; fet-a—fetuin-A; HD—haemodialysis; HF—heart failure; HFmrEF—heart failure with mildly reduced ejection fraction; HFpEF—heart failure with preserved ejection fraction; HFrEF—heart failure with reduced ejection fraction; IVS—Interventricular septal end diastolic dimension; MACEs—major adverse cardiovascular events; MPI—myocardial performance index; NE- norepinephrine; NT-proBNP—N-terminal pro-B-type natriuretic peptide; LVPW—left ventricular posterior wall thickness; RF—rheumatoid factor; SAP—stable angina pectoris; sPESI—Simplified Pulmonary Embolism Severity Index; STEMI—myocardial infarction wit ST-elevation; TG—test group; UAP—unstable angina pectoris.

## Data Availability

Not applicable.

## Abbreviations

The following abbreviations are used in this manuscript:

95% CI	95% confidence interval
ACS	Acute coronary syndrome
ANS	Autonomic nervous system
CAD	Coronary artery disease
CgA	Chromogranin A
CKD	Chronic kidney disease
CTS	Catestatin
CTO	Chronic total occlusion of coronary artery
CVD	Cardiovascular disease
DM	Diabetes mellitus
HA	Arterial hypertension
HF	Heart failure
HR	Hazard ratio
HRV	Heart rate variability
LDL	Low-density lipoproteins
LVP	Left ventricular pressure
MACE	Major adverse cardiovascular events
MI	Myocardial infarction
SCD	Sudden cardiac death
STEMI	Myocardial infarction with ST-segment elevation
UAP	Unstable angina pectoris
VEGF	vascular endothelial growth factor
VF	Ventricular fibrillation
VT	Ventricular tachycardia
